# Chlorine-Functional Silsesquioxanes (POSS-Cl) as Effective Flame Retardants and Reinforcing Additives for Rigid Polyurethane Foams

**DOI:** 10.3390/molecules26133979

**Published:** 2021-06-29

**Authors:** Anna Strąkowska, Sylwia Członka, Karolina Miedzińska, Krzysztof Strzelec

**Affiliations:** Institute of Polymer and Dye Technology, Lodz University of Technology, 90-537 Lodz, Poland; sylwia.czlonka@dokt.p.lodz.pl (S.C.); karolina.miedzinska@dokt.p.lodz.pl (K.M.); krzysztof.strzelec@p.lodz.pl (K.S.)

**Keywords:** rigid polyurethane foams, POSS-Cl, flame retardants, porous composite, mechanical properties, hydrophobicity

## Abstract

The subject of the research was the production of silsesquioxane modified rigid polyurethane (PUR) foams (POSS-Cl) with chlorine functional groups (chlorobenzyl, chloropropyl, chlorobenzylethyl) characterized by reduced flammability. The foams were prepared in a one-step additive polymerization reaction of isocyanates with polyols, and the POSS modifier was added to the reaction system in an amount of 2 wt.% polyol. The influence of POSS was analyzed by performing a series of tests, such as determination of the kinetics of foam growth, determination of apparent density, and structure analysis. Compressive strength, three-point bending strength, hardness, and shape stability at reduced and elevated temperatures were tested, and the hydrophobicity of the surface was determined. The most important measurement was the determination of the thermal stability (TGA) and the flammability of the modified systems using a cone calorimeter. The obtained results, after comparing with the results for unmodified foam, showed a large influence of POSS modifiers on the functional properties, especially thermal and fire-retardant, of the obtained PUR-POSS-Cl systems.

## 1. Introduction

Foams are the main group of polyurethane materials that dominate the market with over 65% of the world’s polyurethane production [1,2]. They are characterized by a porous structure, low apparent density, and high strength. Their properties can be modified by the appropriate selection of components and their mutual ratios [3,4,5]. Polyurethane porous materials fall into two main groups: flexible polyurethane foams and rigid polyurethane foams. One of the most important functional features of foams is the low thermal conductivity coefficient determined by the cell structure of porous materials. The content of open cells in the structure increases the heat transfer capacity [2]. Rigid polyurethane foams are strongly cross-linked materials characterized by a closed-cell structure which affects a high compressive strength and a low thermal insulation coefficient [6,7]. As for the application, due to their good thermal insulation, mechanical and physical properties, and lightweight polyurethane foams are generally used as a low-cost material widely used in building insulation, transportation, electronics, packaging, furniture, and others [5,8]. In addition, flexible polyurethane foams show excellent sound-absorbing properties and the ability to damp vibrations, which allows them to be used as acoustic materials [9].

The main drawback of PUFs is their easy ignition and high flame spreadability, which makes their use limited in many engineering applications [10]. It is known that the degradation of the urethane bonds of rigid polyurethane foams begins at 200 °C [11]. However, it is possible to reduce the combustibility of polyurethane foams by applying various flame retardants [12]. There are various types of flame retardants, including: phosphorous compounds [13], bromine compounds [14], melamine compounds [15], expandable graphite [14,16], and inorganic salts [17]. Moreover, inorganic metal oxides and hydroxides including compounds of magnesium, silicon, and aluminum also play an important role in reducing combustibility [18,19,20,21]. Halogen compounds have also been used previously as flame retardants. However, due to the increasing environmental requirements in recent times, their application is limited because of the release of toxic and harmful gases from their combustion [5,22].

Recently, there has been an increasing amount of research into a multifunctional group of flame retardants, polyhedral oligomeric silsesquioxanes (POSSs), that combine characteristics of organic and inorganic materials [2,23]. Their synthesis dates back to the 1950s, but in recent years they have gained growing interest as hybrid organic/inorganic precursors [24]. They are reactive nanofillers with inorganic nanocage ((RSiO1.5)n) surrounded by at most eight organic groups [25]. Polyhedral oligomeric silsesquioxanes can exist in various forms, depending on the spatial isomerism and the type of substituents (alkyl, aryl, or halogenated groups). Scientists discovered that the fireproof effect of these nanofillers is based on the ability to form a ceramic protective layer that reduces the amount of heat released during the pyrolysis and combustion of the material [18,26,27,28]. However, the organic functional groups in the POSS cage are of great importance for the fireproofing properties, as they break the Si-C bond at a temperature of about 300–350 °C in the air. Immediately after this process, POSS cages are fused to form a thermally insulating and oxidation-resistant layer of carbonized silicon carbide [29,30].

When considering the effect of POSS on flame retardancy, two main types of these nanofillers are distinguished: containing eight identical R groups or seven R groups and one functional group R’ which can be an alcohol, ester, epoxy, isocyanate, amine, or silane. The wide range of these substituents enables their selective selection to achieve compatibility of dispersed nanofillers with the polymer matrix. As with the use of other fillers, the incorporation of POSSs into a polymer matrix can affect both the melt viscosity, mechanical strength, and thermomechanical and electrical properties of the polymers [31]. Moreover, polyhedral oligomeric silsesquioxanes can reduce the amount of heat, smoke and CO released during combustion, thereby increasing thermal stability and mitigating fire hazards [18,24,32,33]. It can be concluded that due to their properties POSS based compounds are commonly used in multi-functional applications. Furthermore, thanks to the presence of both reactive and non-reactive groups, POSSs can be readily attached to the polymer matrix by mixing, grafting, and copolymerization [3,34].

## 2. Results and Discussion

### 2.1. The Impact of POSS-Cl on Polyol Viscosity and Processing Parameters of Polyol Premixes

Polyol premixes are typical non-Newtonian shear-thinning fluids for which the viscosity decreases with increasing shear rate. The viscosity of the polyol premixes is a parameter that determines the rate of foam growth. Therefore, it is important that the viscosity is not too high due to the addition of solid excipients, as this would drastically limit the foam growth process, resulting in a composite with a very small pore size and high density.

As expected, the addition of POSS particles increased the viscosity of the polyol (Table 1), with the POSS-Ar-Et-Cl (Table 5) containing system having the highest viscosity. In all premixes the dependence of viscosity decrease as a function of shear rate was visible. The incorporation of solid particles can not only disrupt the porous structure as a result of an increase in viscosity but also, depending on the reactivity of the surface, can create a risk of aggregates and agglomerates forming in the polymer matrix. This phenomenon is very undesirable as it can have a negative effect on the mechanical properties or the insulating properties. Therefore, it is important to choose the modifier so that it disperses well in the reaction mixture and that it does not have a tendency to aggregate.

The polymerization reaction of PUR foams is a strongly exothermic reaction with the release of a large amount of heat. For the reference foam, the maximum temperature was 118.2 °C. On the other hand, the introduction of POSS-Cl significantly increased the reactivity of the system, which was manifested by an increase in the maximum temperature during foam synthesis. The largest increase in Tmax was recorded for the PUR-POSS-Ar-Et-Cl system (141.2 °C). In addition, the individual times of foam synthesis have also changed. Due to the higher viscosity value, the initial foaming was slower, which was manifested in the longer creaming time of the composition with the addition of POSS. Compared to PUR-0, creaming time increases from 40 s to more than 45 s. However, well-dispersed solids can act as an additional nucleation center, leading to the formation of more vesicle cells during the nucleation process, which could be manifested by accelerating foam expansion, where the rising time shortened from 270 s for PUR-0 to even 215 s for PUR-POSS-Pr-Cl. The introduction of the modifier also leads to a shortening of the tack-free time. This means that the POSS-Cl particles can act as a hardening accelerator during the foaming process.

### 2.2. The Impact of POSS-Cl on Morphology and Apparent Density PUR Foams

**Figure 1:**
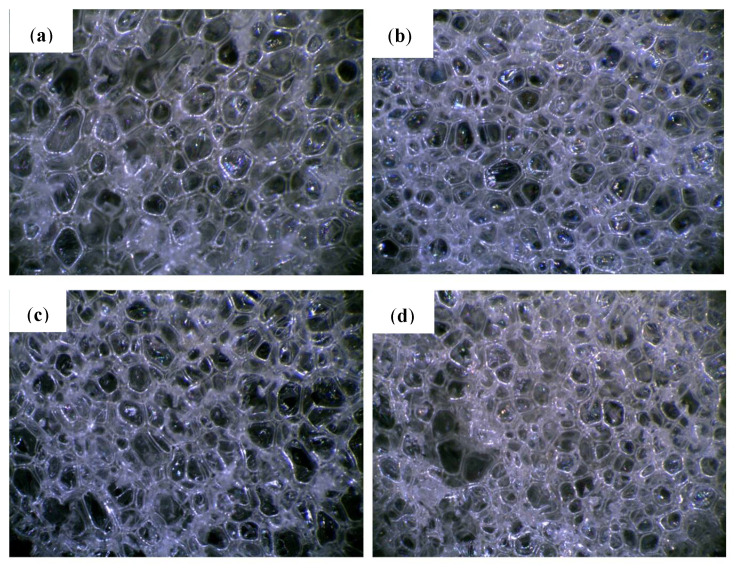
Cellular morphology of (**a**) PUR-0, (**b**) PUR-POSS-Ar-Cl, (**c**) PUR-POSS-Pr-Cl and (**d**) PUR-POSS-Ar-Et-Cl.

The cellular structure is an important parameter of porous materials, determining their mechanical and thermal properties. Figure 1 shows that PUR-0 has the most regular pore size and distribution. The shape of the PUR foam cells was typically a closed polyhedron ranging in size from 410 to 475 µm, and the number of open or damaged cells is small. Microscopic photos of the foams with the addition of POSS show that their cell sizes are less uniform than in the case of the reference foam. The number of open or damaged cells has increased and a larger dispersion of pore size can be observed. Presumably, POSS particles can act as nucleation centers, leading to the formation of smaller cells [35]. As shown in previous studies, particulate matter changes the rheology of the system and reduces the nucleation energy. The lowered nucleation barrier facilitates the extensive formation of smaller cells which later fuse into larger cells [36].

**Figure 2:**
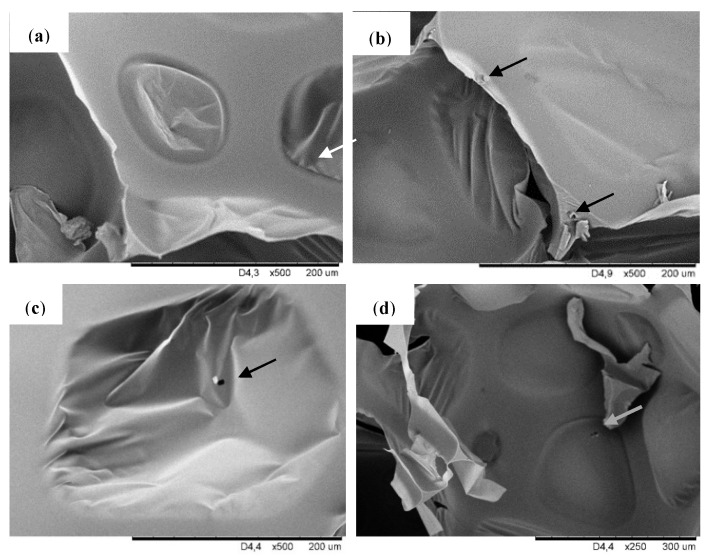
SEM images of (**a**) PUR-0, (**b**) PUR-POSS-Ar-Cl, (**c**) PUR-POSS-Pr-Cl and (**d**) PUR-POSS-Ar-Et-Cl.

Figure 2 shows the SEM pictures taken at a higher magnification, where the filler particles dispersed in the pores of modified foams can be observed. The filler particles can exist both in the cell struts as well as inside the pores. It is visible that for modified foams particles are attached to the cell wall and cell struts which is visible for foam PUR-POSS-Ar-Et-Cl and PUR-POSS-Pr-Cl. In contrast, in the case of foam PUR-POSS-Ar-Cl, filler particles are observed in the void cell space.

One of the most important parameters determining the use of PUR foams as a thermal insulation material is the apparent density, which indirectly affects the mechanical properties, such as compressive strength and hardness. Changes in the structure as a result of the introduction of POSS are manifested by an increase in the apparent density of foams (Table 2). The reason for this slight increase may be the higher initial viscosity of the polyol premix due to the implementation of POSS, which also affects the heterogeneous nucleation process and the formation of more small cells. However, taking into account that the density of polyurethane foam is very sensitive to slight changes in environmental conditions, such as humidity, temperature, or slight changes in mixing time, it can be concluded that the differences in density are not too large [37].

### 2.3. Mechanical Properties of PUR Foams

**Figure 3:**
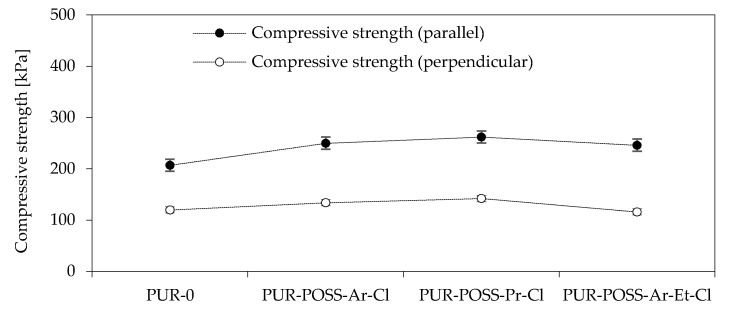
Compressive strength in the direction parallel and perpendicular to the growth direction of PUR foams.

To determine the mechanical properties of the PUR foam, the compressive strength at 10% deformation was tested. Since in rigid PUR foams there is anisotropy of cells manifested by cell elongation in the direction of foam growth, the samples were tested in two directions: parallel and perpendicular to the direction of foam growth (Figure 3). By analyzing the data from the diagram, it can be seen that the addition of POSS-Cl has a significant effect on increasing the compressive strength of PUR. The PUR-0 foam showed a compressive strength parallel to the direction of the foam growth of 207 kPa, while for modified foams there is a tendency to increase the mechanical strength to even 262 kPa for PUR-POSS-Pr-Cl. This 25% increase in strength was due to the higher foam density resulting from the introduction of the POSS cage structure. For other modified foams, a positive effect on strength parameters is also visible. Much lower strengths were obtained in the case of measurements carried out in the direction perpendicular to the direction of foam growth. But also in this case, the introduction of POSS-Cl improved the compressive strength. Only a slight deterioration was noted for PUR-POSS-Ar-Et-Cl, which could be due to the fact that this foam was characterized by the most heterogeneous structure.

**Figure 4:**
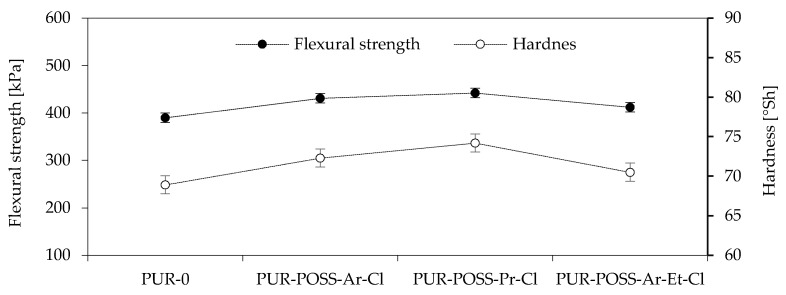
Flexural strength and Hardness of PU foams.

The obtained results of the highest bending stress transmitted by the sample (δfm), called the flexural strength, are shown in Figure 4. Based on the results, it can be seen that the addition of POSS-Cl also significantly changed the flexural strength of the foams obtained. For all modified foams, an increase in the value of the maximum bending stress transferred by the sample was observed. The PUR-POSS-Pr-Cl series is characterized by the highest bending resistance, for which δfm is equal to 442 kPa. This result is higher by almost 15% compared to PUR-0. A very similar tendency can be seen in the case of foam hardness results. The increase in hardness was also caused by the higher density of the samples, although the standard deviation for these results was quite large, which resulted from the heterogeneity of the structure and the large dispersion of the obtained results. Therefore, it should be remembered that the addition of modifiers disrupts the cellular structure, which may cause deterioration of mechanical properties; therefore, it is important to properly select the amount of the introduced modifier.

### 2.4. Hydrophobic Properties of PUR Foams

**Figure 5:**
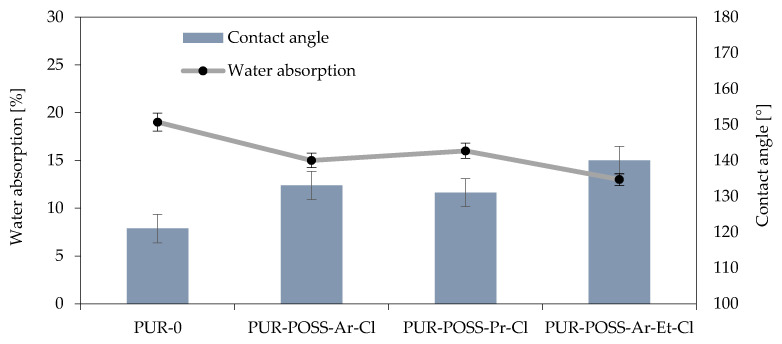
Effect of contact angle on water absorption of RPUFs modified with POSS-Cl.

The presence of Cl in the POSS particle resulted in its low surface energy, which could translate into a reduction in the hydrophobicity of the entire system [38]. Therefore, the water absorption and contact angle of the foams’ surfaces were measured for the samples (Figure 5). These parameters are important from the operational point of view because it is important that insulation materials made of PUR do not show a tendency to absorb water or moisture from the environment, which could change their general performance parameters and lead to faster degradation.

The measurement of short-term water absorption showed a positive effect on the reduction of water absorption by foam. In all modified foams, it was possible to reduce the water absorption, where the use of POSS-Ar-Et-Cl allowed for a 30% reduction in this parameter. This effect was caused not only by the nature of the halide POSS themselves but also by the more irregular structure which also has a strong influence on the hydrophobicity of the surface.

The reduction in affinity for water is also well illustrated by the results of the measurements of the contact angle of the foam surfaces, which show that the foams with the addition of POSS-Cl are characterized by much lower wettability by water compared to PUR-0. This test also confirmed the most hydrophilic nature of the sample with the addition of POSS-Ar-Et-Cl, for which a contact angle of 140° was obtained (for the PUR-0 sample, the contact angle was 121°).

### 2.5. Thermal Stability and Fire Behavior of PUR Foams

**Figure 6:**
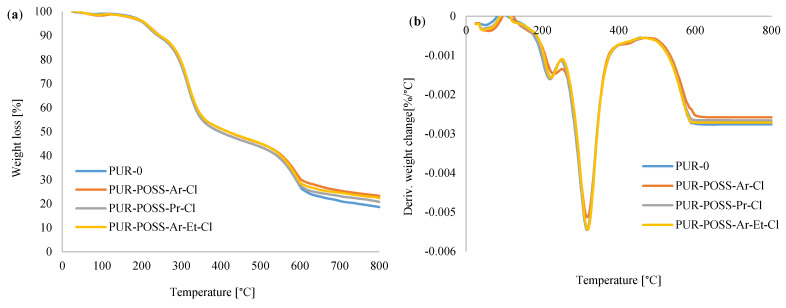
(**a**) TGA and (**b**) DTG curves for PUR foams modified with POSS-Cl.

To evaluate the influence of the applied flame retardants on thermal stability, TGA measurements were performed. Figure 6a,b shows the thermogravimetric (TGA) and derivative thermogravimetric (DTG) curves of analyzed foams. Thermal degradation of RPUFs takes place in three stages. The first stage of decomposition occurs from about 150 to 250 °C and corresponds to about 10% weight loss. It is associated with the dissociation of the urethane bonds corresponding to the degradation of the hard segments [8,11]. The second stage of degradation, manifested by about 50% loss of initial mass starts between 300 and 350 °C and it corresponds to the thermal decomposition of the soft polyol segments of PUR [39]. The last, third degradation stage, in which the weight loss reaches about 70%, occurs between 500 and 600 °C and it is related to the decomposition of the fragments formed in the previous stage into volatile products [40].

Table 3 shows the characteristic temperature values corresponding to the successive stages of decomposition. T_5_ is the temperature corresponding to a 5% weight loss, similarly T_10_, T_50_ and T_70_ corresponding to 10, 50 and 70% weight loss to the initial mass of samples. Comparing the foams containing the POSS additives with the reference PUR-0 foam, it has been noticed that modified PUR foams required a higher temperature to pass between the degradation steps. The results summarized in the table show that the PUR-POSS-Pr-Cl foam reached temperatures slightly higher than the reference sample during the subsequent stages of degradation. On the other hand, PUR-POSS-Ar-Cl and PUR-POSS-Ar-Et-Cl samples show increased thermal stability, which is especially noticeable in the analysis of weight loss over 50%.

The determination of char residue by TGA is a relative method of assessing the fire resistance of a polymer [41]. The foams containing the addition of modifiers showed a higher percentage of carbonation residues than the reference foam, where at 800 °C PUR-POSS-Ar-Cl, PUR-POSS-Pr-Cl and PUR-POSS-Ar-Et-Cl the carbon content increased, respectively, up to 23.2, 20.8 and 22.4% compared to 18.6% for the PUR-0 reference sample. It can be concluded that POSS-Cl can act as a charring agent, which positively influences not only the thermal stability, but also the fire resistance of PUR foams. All these observations may indicate the effect of POSS additives on increasing thermal stability.

The fire resistance properties of PUR based on combustion in a cone calorimeter are represented by the ignition time (TTI), the peak heat release rate (pHRR), and total smoke release (TSR), total heat release (THR), the maximum average rate of heat emission (MAHRE) as well as the total smoke release (TSR) and char residue.

Analyzing the data in Table 4, characterizing the combustion process of PUR foams, a positive effect of POSS-Cl on the reduction of flammability of modified foams can be noticed. TTI for PUR-POSS-Cl is longer than for pure PUR-0. In addition, the parameter informing about the ignition spark of the combusted material was reduced, i.e., the heat release rate—determined by the pHRR. Namely, for the samples PUR-POSS-Ar-Cl, PUR-POSS-Pr-Cl and PUR-POSS-Ar-Et-Cl, the pHRR value was 172, 211, 189 kW/m^2^, respectively, compared to 268 kW/m^2^ for the reference sample. A similar trend was observed for MARHE. Such an improvement in the fire resistance of PUR composite foams may be related to the formation of a protective carbon layer in the initial decomposition phase, which is an insulating barrier for the material beneath it and prevents the transfer of necessary heat and gases for further combustion. The carbon layer can also reduce the amount of smoke and harmful gases released during combustion. It is also believed that POSS may migrate to the surface during initial ignition and be degraded by homolytic breakage of Si-C bonds. The resulting ceramic layer consisting of stable Si-O bonds creates a reinforced carbon layer, which protects the material against further combustion [42]. In addition, the combustion reactions are radical, and the chlorine atoms formed during the decomposition of POSS act as radical scavengers, additionally inhibiting the combustion of the composite.

**Figure 7:**
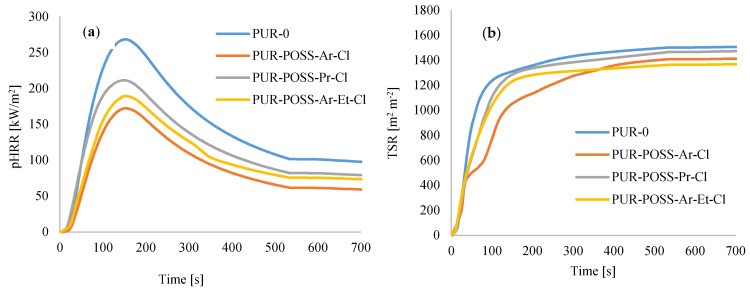
The results of the cone calorimeter test—(**a**) the peak rate of heat release (pHRR), (**b**) the total smoke release (TSR).

Among all series of PUR composite foams, the greatest improvement was observed for PUR composite foams containing POSS with aromatic structures (Figure 7a,b)—the pHRR value decreased by about 30%. Such improvement is a result of the stabilization of the aromatic system by the delocalization of the pi-electrons on the ring [43]. In addition, for all modified PUR-POSS-Cl foams lower TSR values were recorded, which additionally increases safety during a fire, as smoke poisoning during a fire is as dangerous as contact with fire or high temperature. Moreover, the introduction of POSS-Cl slightly lowers the value of total heat release (THR).

## 3. Materials and Methods

### 3.1. Materials

The water-blown RPUFs used in this study were obtained from a two-component system supplied by Purinova Sp. z o. o., after mixing the polyol (Izopianol 30/10/C) and the diphenylmethane diisocyanate (Purocyn B). The polyol is a mixture of components containing polyester polyol (hydroxyl number ca. 230−250 mgKOH/g, functionality of 2), catalyst (*N*,*N*-Dimethylcyclohexylamine), flame retardant (Tris(2-chloro-1-methylethyl) phosphate), a chain extender (1,2-propanediol) and water as a blowing agent. PUR foams were modified with silsesquioxanes functionalized with halogen: chloropropylisobutyl-POSS (POSS-Pr-Cl), chlorobenzylisobutyl-POSS (POSS-Ar-Cl), chlorobenzylethylisobutyl-POSS (POSS-Ar-Et-Cl) from Hybrid Plastics, Inc (Hattiesburg, MS, USA). Their structural formulas are shown in Table 5. The POSS-Cl modifiers were added to the reaction system in an amount of 2 wt.% relative to the weight of the polyol.

### 3.2. Synthesis of PUR Composites Foams

PUR composite foams were produced using the method described in the previous works. Briefly, the synthesis of PUR composite foams modified with POSS-Cl was as follows: polyol premix (Izopianol) was placed in a plastic cup and intensively mixed at 1000 rpm with a mechanical mixer for 60 s. The appropriate amount of POSS-Cl modifier was then added to the cup and mixed for another 60 s to obtain a homogeneous dispersion. The calculated amount of isocyanate (Purocyn) was added to the reaction mixture and thoroughly mixed for 30 s. The reaction mixture was allowed to grow freely by measuring the individual cream, growth and tack-free times, and the maximum reaction temperature (Tmax). The grown PUR foams were left at room temperature for 24 h to ensure complete curing of the composites.

### 3.3. Methods and Instruments

The viscosity of the polyol systems was evaluated using a Viscometer DVII+ (Brookfield, Hadamar-Steinbach, Germany) in the function of a shear rate according to ISO 2555. The measurement was performed at ambient temperature.

The apparent density of the analyzed foams was measured according to the standard ASTM D1622 (equivalent to ISO 845). The density was tested on five samples of each foam and expressed as an average.

Cell size distribution and foam morphology were examined based on the cellular structure pictures of foams taken using JEOL JSM-5500 LV scanning electron microscopy (JEOL LTD, Akishima, Japan). The microscopic research was carried out in a high-vacuum mode and at the accelerating voltage of 10 kV.

A three-point bending test was carried out accordingly to the standard ASTM D7264 (equivalent to ISO 178) using a Zwick Z100 Testing Machine (Zwick/Roell Group, Ulm, Germany). The analyzed samples were bent with a speed of 2 mm min−1. For each series of foams, at least five measurements were made. Obtained flexural stress at the break results for each sample was expressed as a mean value and averaged.

The compressive strength (σ10%) of the foams was determined according to the standard ASTM D1621 (equivalent to ISO 844). The measurement was conducted using a Zwick Z100 Testing Machine (Zwick/Roell Group, Ulm, Germany) with a load cell of 2 kN and a speed of 2 mm min^−1^. The compression strength was examined as a ratio of the load causing 10% deformation of samples cross-section in both parallel and perpendicular direction to the square surface. The compressive strength was measured in five samples of foam (8 cm × 8 cm × 5 cm) and expressed as an average.

Surface hydrophobicity of PUR foams was measured using contact angle goniometer OEC-15EC (DataPhysics Instruments GmbH, Filderstadt, Germany) with software module SCA 20. Water absorption of PU foams was performed according to ISO 2896:2001.

The thermal stability of the foams was analyzed using a Mettler Toledo thermogravimetric analyzer TGA/DSC1. A thermal decomposition examination was conducted in air (flow 50 mL min^−1^) and in the temperature range between 25 and 600 °C (heating rate 10 °C min^−1^). The measurement included an analysis of the mass change as a function of temperature during thermal decomposition of the polyurethane foams. The initial temperatures of the following decomposition stages were noticed and designated as T5%, T10%, T50%. These temperature values corresponded to the percentage of weight loss.

The burning behavior and flame-retardant properties of the foams were analyzed using a cone calorimeter, according to the standard ISO 5660 in S.Z.T.K. TAPS (Maciej Kowalski Company, Saugus, Poland). The measurement for each foam was repeated on three samples and averaged. Each specimen with dimensions of 10 cm × 10 cm × 5 cm was wrapped with aluminum foil and burned at an external heat flux of 35 kW m^−2^. The parameters were recorded during the time.

## 4. Conclusions

The paper presents the effect of POSS-Cl as modifiers of the functional properties of rigid PUR foams, with particular emphasis on the flame-retardant properties of silsesquioxanes. The conducted research shows that the introduction of the silicate cage modifier significantly influences the properties of the tested systems, starting from the rheological properties of the polyol premixes, which resulted in the subsequent characteristics of the obtained PUR-POSS-Cl foams. Starting from a change in structure to a more heterogeneous one with smaller and more irregular cells, they simultaneously resulted in a higher density of the molded foams. This, in turn, translated into an increase in the mechanical properties and hardness of the modified PUR foams. It was found that the addition of POSS-Cl had a significant effect on increasing the hydrophobicity of the system as compared to the PUR-0 reference sample. However, the most important aspect of the presented research is the improvement of thermal stability and reduction of flammability of modified PUR-POSS-Cl systems. Among all the silsesquioxanes used, POSS-Ar-Cl can be indicated as the best, as it had the greatest impact on increasing thermal and mechanical properties.

## Figures and Tables

**Table 1:** The impact of POSS-Cl on viscosity and growth kinetic of polyol premixes.

Sample Code	Dynamic Viscosity *η* [mPa·s]			T_Max_ [°C]	Processing Times [s]		
	0.5 RPM	50 RPM	100 RPM		Cream Time	Growth Time	Tack-Free Time
PUR-0	840 ± 9	430 ± 7	320 ± 8	118.2 ± 4	40 ± 2	270 ± 9	365 ± 9
PUR-POSS-Ar-Cl	1120 ± 10	930 ± 9	440 ± 10	135.7 ± 5	47 ± 1	260 ± 11	325 ± 9
PUR-POSS-Pr-Cl	1090 ± 10	870 ± 10	420 ± 11	138.2 ± 4	45 ± 2	215 ± 10	298 ± 12
PUR-POSS-Ar-Et-Cl	1210 ± 10	960 ± 11	470 ± 12	141.2 ± 6	48 ± 2	230 ± 9	314 ± 8

**Table 2:** Parameters of the structure of PUR composite foams.

Sample Code	Cell Size [µm]	Wall Thickness [µm]	Apparent Density [kg m^−3^]
PUR-0	475 ± 10	63 ± 4	39 ± 1
PUR-POSS-Ar-Cl	442 ± 8	68 ± 5	42 ± 2
PUR-POSS-Pr-Cl	421 ± 9	67 ± 2	43 ± 2
PUR-POSS-Ar-Et-Cl	413 ± 12	69 ± 3	41 ± 2

**Table 3:** Results of thermogravimetric analysis of PUR foams modified with POSS-Cl.

Sample Code	T_5_ [°C]	T_10_ [°C]	T_50_ [°C]	T_70_ [°C]	Char Residue [%]
PUR-0	209	241	397	589	18.6
PUR-POSS-Ar-Cl	211	247	419	605	23.2
PUR-POSS-Pr-Cl	207	241	399	593	20.8
PUR-POSS-Ar-Et-Cl	211	245	417	597	22.4

**Table 4:** Results of fire behavior of PUR foams modified with POSS-Cl.

Sample Code	TTI (s)	pHRR (kW/m^2^)	THR (MJ/m^2^)	MAHRE (kW/m^2^)	TSR (m^2^/m^2^)	Char Residue [%]
PUR-0	2 (0)	268 (8)	21.4 (1)	168 (8)	1490	18.9 (3)
PUR-POSS-Ar-Cl	5 (1)	172 (11)	20.2 (2)	128 (13)	1411	21.4 (5)
PUR-POSS-Pr-Cl	4 (0)	211 (13)	20.6 (2)	153 (16)	1472	19.6 (4)
PUR-POSS-Ar-Et-Cl	4 (0)	189 (13)	20.5 (3)	139 (11)	1367	18.2 (4)

**Table 5:** POSS-Cl compounds used for modification of PUR foams.

Coumpound	Abbreviation in the Text	Structure	Summary Formula
Chlorobenzyllsobutyl POSS	POSS-Ar-Cl	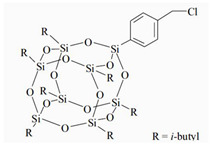	[C_35_H_69_ClO_12_Si8]
Chloropropyllsobutyl POSS	POSS-Pr-Cl	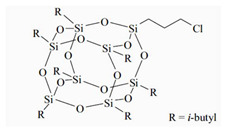	[C_31_H_69_ClO_12_Si_8_]
Chlorobenzylethyllsobutyl POSS	POSS-Ar-Et-Cl	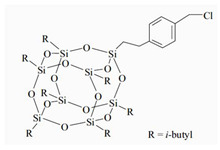	[C_37_H_73_ClO_12_Si_8_]

## Data Availability

The data presented in this study are available on request from the corresponding author.
